# Infection of porcine small intestinal enteroids with human and pig rotavirus A strains reveals contrasting roles for histo-blood group antigens and terminal sialic acids

**DOI:** 10.1371/journal.ppat.1009237

**Published:** 2021-01-29

**Authors:** Yusheng Guo, Rosario Adriana Candelero-Rueda, Linda Jean Saif, Anastasia Nickolaevna Vlasova

**Affiliations:** Food Animal Health Research Program, Ohio Agricultural Research and Development Center, Department of Veterinary Preventive Medicine, The Ohio State University, Wooster, Ohio, United States of America; Instituto de Biotecnologia, MEXICO

## Abstract

Rotaviruses (RVs) are a leading cause of acute viral gastroenteritis in young children and livestock worldwide. Growing evidence suggests that host cellular glycans, such as histo-blood group antigens (HBGAs) and sialic acids (SA), are recognized by the RV surface protein VP4. However, a mechanistic understanding of these interactions and their effects on RV infection and pathogenesis is lacking. Here, we established a *p**orcine* crypt-derived *3D* *i**ntestinal* *e**nteroids (PIEs) culture* system which contains all intestinal epithelial cells identified in vivo and represents a unique physiologically functional model to study RV-glycan interactions in vitro. PIEs expressing different HBGAs (A+, H+, and A+/H+) were established and isolation, propagation, differentiation and RV infection conditions were optimized. Differentiated PIEs were infected with human RV (HRV) G1P[8] Wa, porcine RV (PRV) G9P[13], PRV Gottfried G4P[6] or PRV OSU G5P[7] virulent and attenuated strains and virus replication was measured by qRT-PCR. Our results indicated that virulent HRV G1P[8] Wa replicated to the highest titers in A^+^ PIEs, while a distinct trend was observed for PRV G9P[13] or G5P[7] with highest titers in H^+^ PIEs. Attenuated Wa and Gottfried strains replicated poorly in PIEs while the replication of attenuated G9P[13] and OSU strains in PIEs was relatively efficient. However, the replication of all 4 attenuate strains was less affected by the PIE HBGA phenotypes. HBGA synthesis inhibitor 2-F-Peracetyl-Fucose (2F) treatment demonstrated that HBGAs are essential for G1P[8] Wa replication; however, they may only serve as a cofactor for PRVs G9P[13] and OSU G5P[7]. Interestingly, contrasting outcomes were observed following sialidase treatment which significantly enhanced G9P[13] replication, but inhibited the growth of G5P[7]. These observations suggest that some additional receptors recognized by G9P[13] become unmasked after removal of terminal SA. Overall, our results confirm that differential HBGAs-RV and SA-RV interactions determine replication efficacy of virulent group A RVs in PIEs. Consequently, targeting individual glycans for development of therapeutics may not yield uniform results for various RV strains.

## Author summary

Cell surface glycans, including histo-blood group antigens (HBGA) and sialic acids (SAs), have been shown to serve as receptors/attachment factors for many pathogens including RVs. However, how those glycans affect RV replication remains largely unknown due the lack of reliable in vitro models. To solve this problem, we established a 3D porcine intestinal enteroid (PIE) model that recapitulates the complex intestinal morphology better than conventional cell lines. By utilizing PIEs expressing different types of HBGAs, we found that several RV strains including Wa G1P[8], OSU G5P[7] and G9P[13] show preference for certain HBGA types. Interestingly, only Wa replication was reduced when HBGAs synthesis was inhibited, while that of OSU and G9P[13] was only marginally affected, which indicates that they may utilize alternative attachment factors for infection. Sialidase treatment strongly inhibited the growth of OSU, while G9P[13] replication was significantly enhanced. These findings suggest that SAs play contrasting roles in the infection of PRV OSU and G9P[13] strains. Overall, our studies demonstrate that PIEs can serve as a model to study pathogen-glycan interactions and suggest that genetically distinct RVs have evolved diverse mechanisms of cell attachment and/or entry.

## Introduction

Rotaviruses (RVs) are an important cause of severe diarrheal illness in infants and young animals including pigs [1]. In children, RVs cause approximately $96 million in annual costs due to hospitalizations in the United States [2,3]. In addition, RVs are responsible for ~7–50% mortality in piglets, resulting in major economic losses to the pork industry[4]. RVs are non-enveloped double-strand RNA (dsRNA) viruses. RVs are classified into ten antigentically and genetically distinct groups from A-J, designed as RVA, RVB, RVC, RVD, RVE, RVF, RVG, RVH, RVI and RVJ respectively [5–7]. RVAs were thought to be the most prevalent and pathogenic among the ten groups [8]; however, recent data has demonstrated increased prevalence and pathogenicity of RVB, RVC and RVH in humans and pigs [9–11]. Within each group, RVs are further classified into G and P genotypes based on molecular characteristics of their outer surface glycoprotein VP7 and the protease-sensitive spike protein VP4 (cleaved into VP8* and VP5* under protease treatment), respectively [8,12].

A substantial body of information on RV host cell attachment has been generated RV during the past 20 years. However, the entry mechanism of RV is not yet fully understood. Although the actual cellular receptors of RVs are not confirmed, early studies indicated that the VP4 protein ([P] type) is associated with the initial interaction of RV and sialic acid (SA) [13,14], and the neuraminidase (sialidase) sensitivity assay showed that only a few P genotypes of animal RVs (e.g., porcine OSU and simian RRV) are sialidase-sensitive strains, while most of the animal and human RVs (e.g., strains Wa and DS1) are SA independent [13,15,16]. These studies also prompted a search for additional receptors or attachment factors for those sialidase-insensitive RV strains.

**Fig 1:**
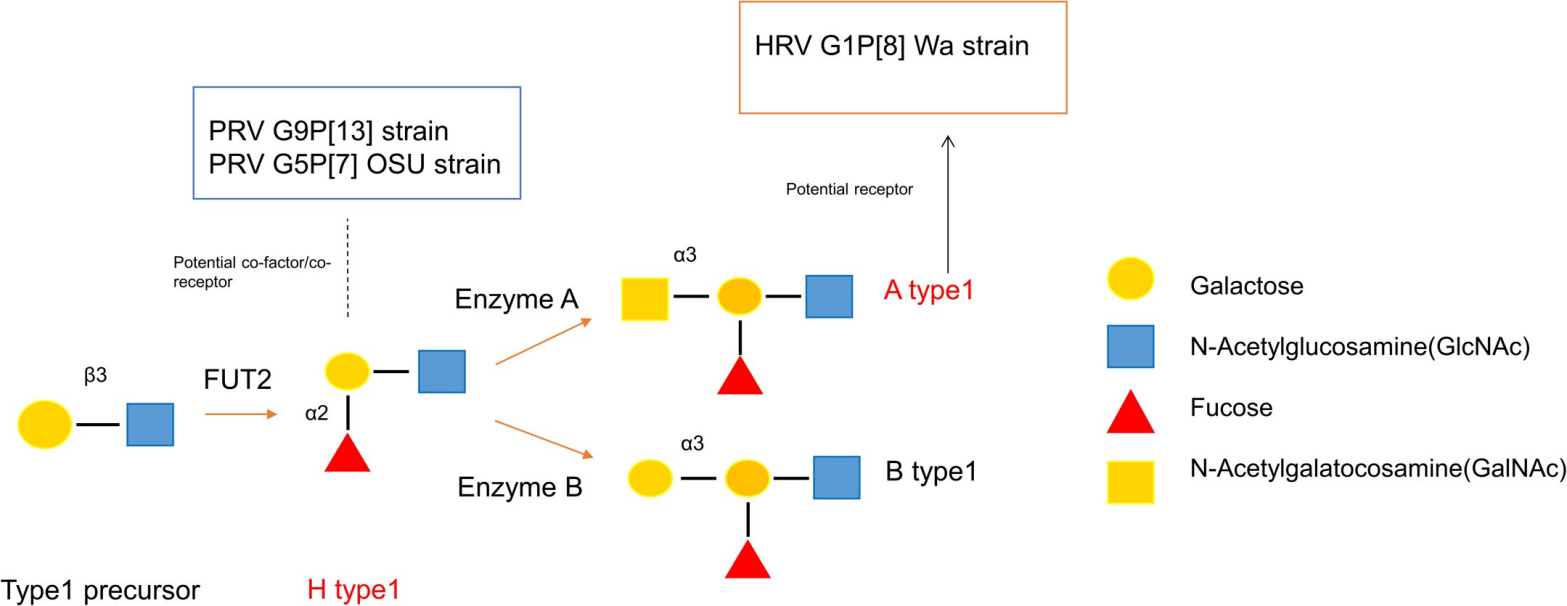
Biosynthesis of Type 1 HBGAs. HBGAs are synthesized by sequential addition of a monosaccharide to a precursor glycan, catalyzed by different enzymes. FUT2 catalyzes the synthesis of secretor antigens through the linkage-specific addition of a-fucose to the galactose and forms the H type1 antigens. H type1 antigens could be processed further by adding a N-Acetylgalatocosamine (GalNAc) or a galactose through a3 linkage to form A or B antigen which are catalyzed by enzyme A or enzyme B, respectively.

Subsequent studies suggested that the VP8* of a human RV strain HAL1116 (P[14]) selectively recognizes the type A histo-blood group antigen (HBGAs), highlighting the importance of interactions between RVs and HBGAs [17,18]. Further, HBGAs are also known to act as attachment (co-)receptors/(co-)factors for multiple pathogens including noroviruses [19] and coronaviruses [20]. HBGAs are complex glycans present on the surface of many cell types, including red-blood cells (RBCs), gut epithelial cells [21] and as free molecules in mucosal secretions and body fluid such as saliva and milk in secretor positive individuals [22]. Biosynthesis of HBGAs starts with generation of the type 1 precursor (Galβ1-3GlcNAcβ-R) which can be converted into different antigens by step-wise addition of saccharide residues during each enzymatic catalyzation step (Fig 1) to become ABH(O), Lewis and secretor/non-secretor types [22,23]. This process is developmentally regulated in children and is hypothesized to be associated with age-specific susceptibility to RV infection [24]. Moreover, human HBGAs share homology with those of many animals including swine (major type is A and H type1) and the similarities between human and animal HBGAs could form the basis for a mechanism of zoonotic and interspecies transmission of RVs. Thus, elucidating the interaction between RVs, including attenuated strains and HBGA may increase our knowledge regarding RV attachment, zoonotic transmission and vaccine efficacy [24].

Although significant efforts have been made to dissect the mechanisms of the RV-HBGA interactions, many discrepancies are found in recent studies. An *in vitro* binding assay indicated that strains of P[4] and P[8] genotypes reacted with H type 1 and Lewis b (Le^b^) antigens, while strains of P[6] bound to H type 1 only [25]. However, in contrast to the above finding, results of a nuclear magnetic resonance (NMR) solution study suggested that the major human RVs including P[4], P[6], P[9], P[14], but not P[8] recognize A type HBGA [26]. In addition, the latter study showed that none of the RV strains tested recognized H-type-1 and Le^b^ antigens. Interestingly, an X-ray crystallography-based investigation showed that a conserved binding site was present in all tested P[4], P[6], P[8] strains which would recognize the type1 precursor and support the interactions of VP8* with ABH antigen, but likely would be unable to recognize Lewis HBGA [12,27]. While some studies are in support of these findings, others contradict them. There are several possible explanations for the observed discrepancies on the RV-HBGA interaction are. 1) Different methodology used in each study. Glycan binding assay [25], NMR solution [26] and X-ray crystallography[12] were applied to analyze the VP8* structure and its binding to HBGA antigens, respectively. 2) The lack of a suitable, physiologically relevant in vitro model. Although infectivity assays have been performed in some of the studies [12,26,28], cell lines that do not express HBGAs or transformed cell lines (e.g. HT-29) were used in these studies. Using conventional cell lines would not be representative of the in vivo RV-HBGA interactions because 2D cultured cell lines usually fail to recapitulate the morphology and physiology of the small intestine. Thus, an appropriate and robust in vitro model is required to evaluate the role of the HBGAs during RV infection.

Intestinal enteroids (IEs) are an in vitro, three-dimensional (3D) culture system that recapitulates the complex nature and functions of the small intestine [29–32]. Crypts isolated from the small intestine are cultured in Wnt3A-rich growth medium and matrigel to support their 3D-structure. The crypt cells give rise to stem, transient amplifying cells and all the small intestinal differentiated epithelial cell lineages [30,33]. Recently, IEs have been demonstrated to have a tremendous potential as a model to study pathogen-host interaction including norovirus, porcine epidemic diarrhea virus (PEDV) and RVs [33–35]. More importantly, IEs naturally express HBGAs which makes them an indispensable in vitro model to investigate the HBGA-RV interaction [36]. Notably, porcine A and H antigens share high homology with those of humans. Differential expression of the porcine A and H antigens can result in four phenotypes, A (or A+), A^w^ (weak A, or A+/H+), O (or H+) or “–” (non-A+H+, or A-/H-)[37]. Because of the shared HBGA antigens and physio-anatomical similarities to humans (Fig 1), pigs are considered an important model to study xenotransplantation [38] and the RV-HBGA interactions in our study.

In the present paper, we have successfully established PIEs that express major HBGAs found in swine (A+, H+, and A+H+) and evaluated the replication levels of several virulent (vir) and attenuated (att) RV strains of common P types in humans or swine. We also identified the roles of HBGAs and SAs in infections by different RV strains.

## Results

### Establishment of PIEs with A+, H+, and A+/H+ HBGA antigens

**Fig 2:**
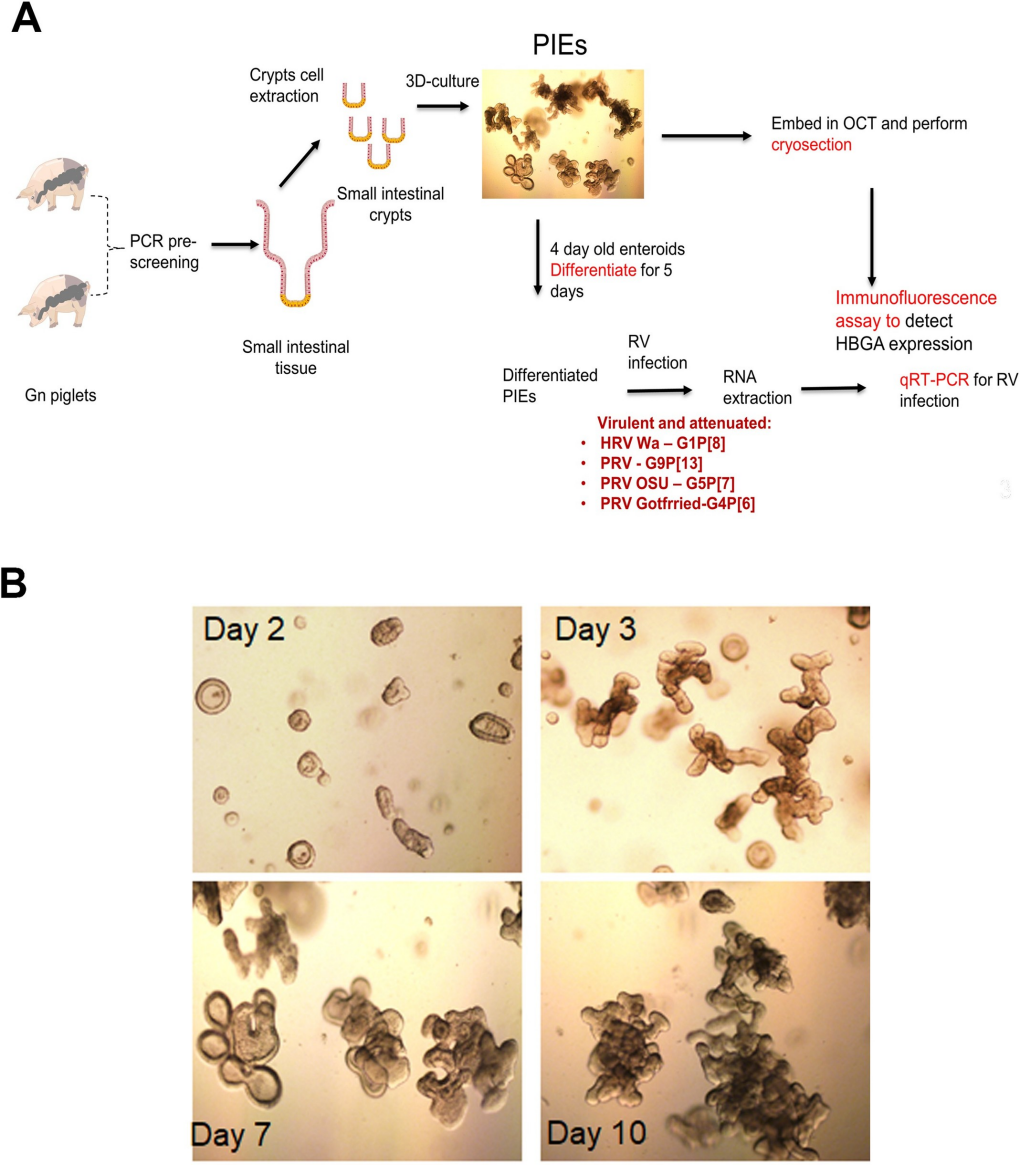
Workflow on PIEs establishment. A) Gn piglets that are 1 week old were pre-screened using PCR methods to detect erythrocyte antigen A (EAA) gene polymorphisms. Crypts cells were extracted from ileum and cultured in IntestiCult medium for several passages to form PIEs. Then, Immunofluorescence (IF) was performed to identify HBGA phenotype and 4-day-old PIEs of each HBGA type were differentiated for 5 days followed by RV infection. B) Morphology of PIEs at different days after passage. Day2, day3, day7 and day10 are represented by the figure in upper left, upper right, lower left and lower right, respectively.

**Fig 3:**
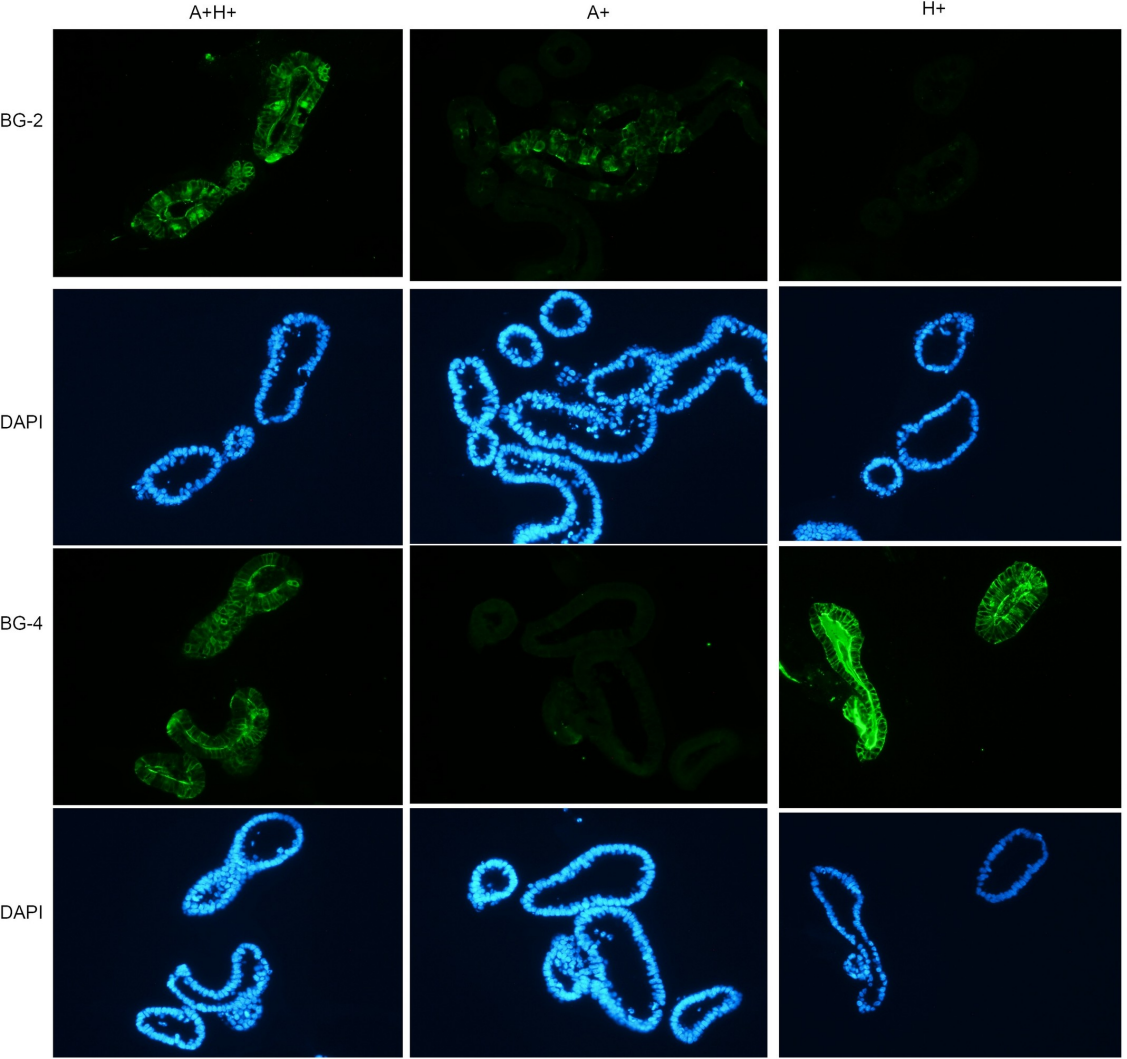
HBGA phenotyping by immunofluorescence antibody. The first row is mouse mAb BG-2 (anti-A antibody) followed by Alexa Fluor 488 Goat anti-mouse IgG3 (1:1000, Thermo Fisher Scientific) stained green. The second row is the corresponding DAPI staining (blue) for the first row. The third row is mouse mAb BG-4 (anti-H antibody) followed by Alexa Fluor 488 Goat anti-mouse IgG3 (1:1000, Thermo Fisher Scientific) stained green and the fourth row is the corresponding DAPI staining for the third row. Thus, the left, middle, and right column represent A+H+, A+, and H+ PIEs, respectively.

Ileal crypts were extracted from Gn piglets that according to pre-screening by PCR/RT-PCR expressed desired HBGAs (Fig 2A and S1 Table). As expected, PIEs grew gradually after establishment and their morphology transitioned from cystic to multi-lobular 3D structures (Fig 2B). Then, the expression of A/H antigens was confirmed using immunofluorescence (IF). We have observed a clear membrane expression pattern (Fig 3) that confirmed that PIEs, of the three major HBGA types (A+, H+ and A+H+) were successfully established.

### Optimization of the medium composition for PIE differentiation

**Fig 4:**
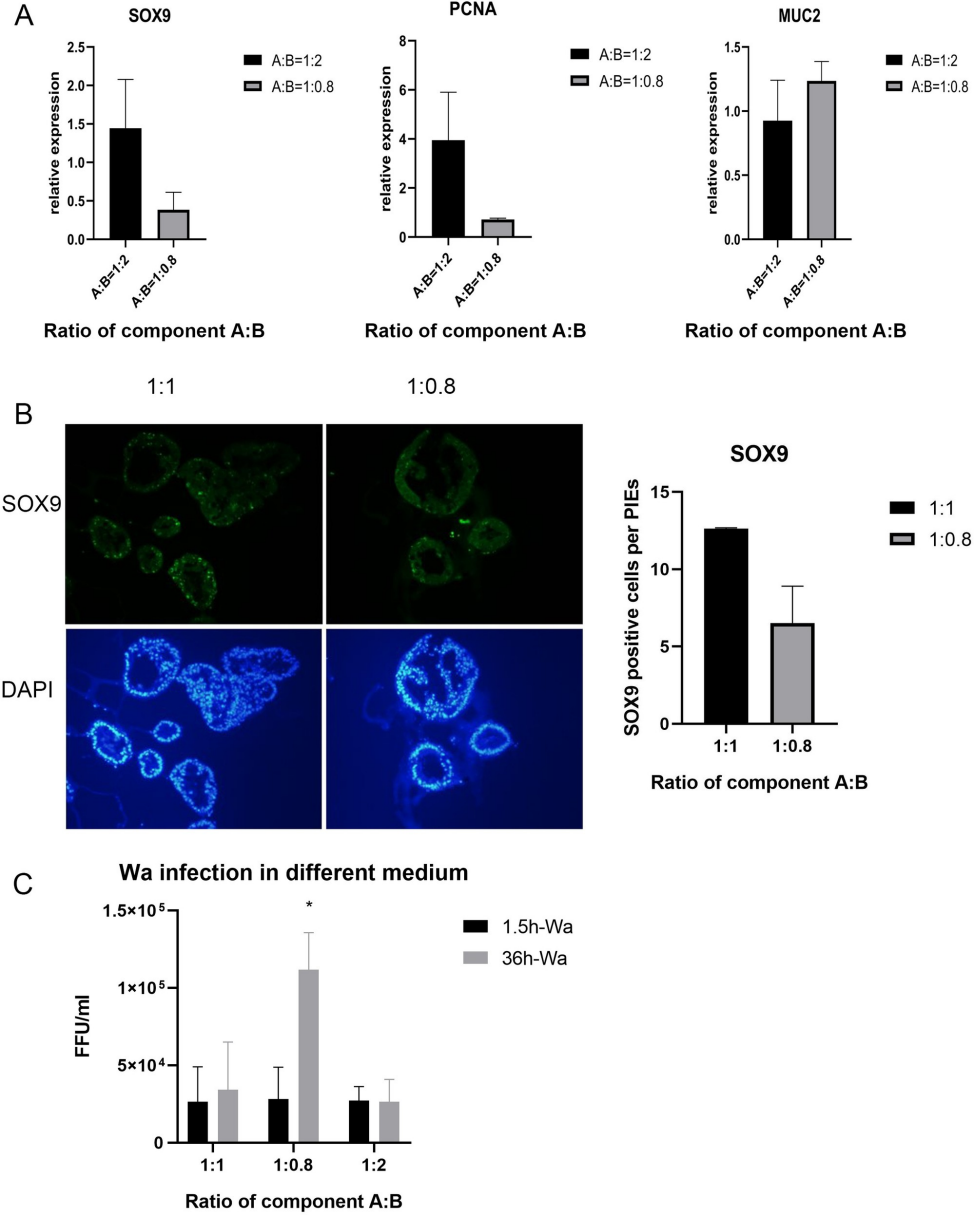
Conditions for PIEs differentiation. Suitable differentiation medium was explored, and several differentiation makers were test under several ratios of component A: B (1:1,1:2,1:0.8). A) RNA expression of SOX9 (stem cell marker), PCNA (cell proliferation marker) and MUC2 (goblet cell marker) were tested by RT-PCR. Figures show the relative expression compared to 1:1 medium (the expression on 1:1 are equal to 1). B) Left panel: IF detection of SOX9 expression. SOX9 (green) and DAPI (blue) staining are shown in the first and second row, respectively. Right Panel: Quantification for the left panel by counting the average numbers of SOX9 positive cells in each PIE. C) Wa infected PIEs cultured in differentiation medium. RV load at 1.5hpi and 36 hpi was examined by RT-PCR. Ct values were converted to FFU/ml based on a standard curve which combined CCIF and RT-PCR results. Error bars are denoted as the standard deviation and all experiments were repeated at least twice. One-way ANOVA was used to compare differences between the 3 groups. For p-value: * p<0.05.

Since terminally differentiated small intestinal enterocytes are the preferred target cells for RVs, we have compared various ratios of components A and B (IntestiCult Organoid Growth Medium) to promote differentiation, while maintaining high viability and proliferative capacity of the PIEs. Because component B of the Complete cell culture medium (CCCM) contains multiple growth factors or signaling inhibitors, the following ratios of the components A and B (1:1, 1:0.8, 1:2, 1:0.5, component A only) were used to incubate PIEs for 4 days. The epithelial cell markers were detected by either RT-PCR (Fig 4A) or IF (Fig 4B). After, culturing PIEs in the medium with 1:0.5 A:B ratio or with component A only (recommended by the manufacturer for human organoids), PIE viability was unsatisfactory, and no further testing was done using this ratio. Further, our results indicate that SOX9 (stem cell marker) and PCNA (cell proliferation marker) expression was decreased in the 1:0.8 medium. By contrast, MUC2 (goblet cell marker) expression was enhanced in 1:0.8 medium. These results suggest that PIEs achieved a higher differentiation status in 1:0.8 medium. To further evaluate the effect of differentiation on RV infection, PIEs were incubated in the medium with the following ratios of components A and B (1:1, 1:0.8, 1:2) followed by HRV Wa infection. Consistent with the above data, the results indicated that Wa replicated to the highest titers in the 1:0.8 medium, referred to as differentiation medium (DM) hereafter (Fig 4C).

### The replication efficiency of RV was affected by HBGA types of PIEs in a strain specific manner

**Fig 5:**
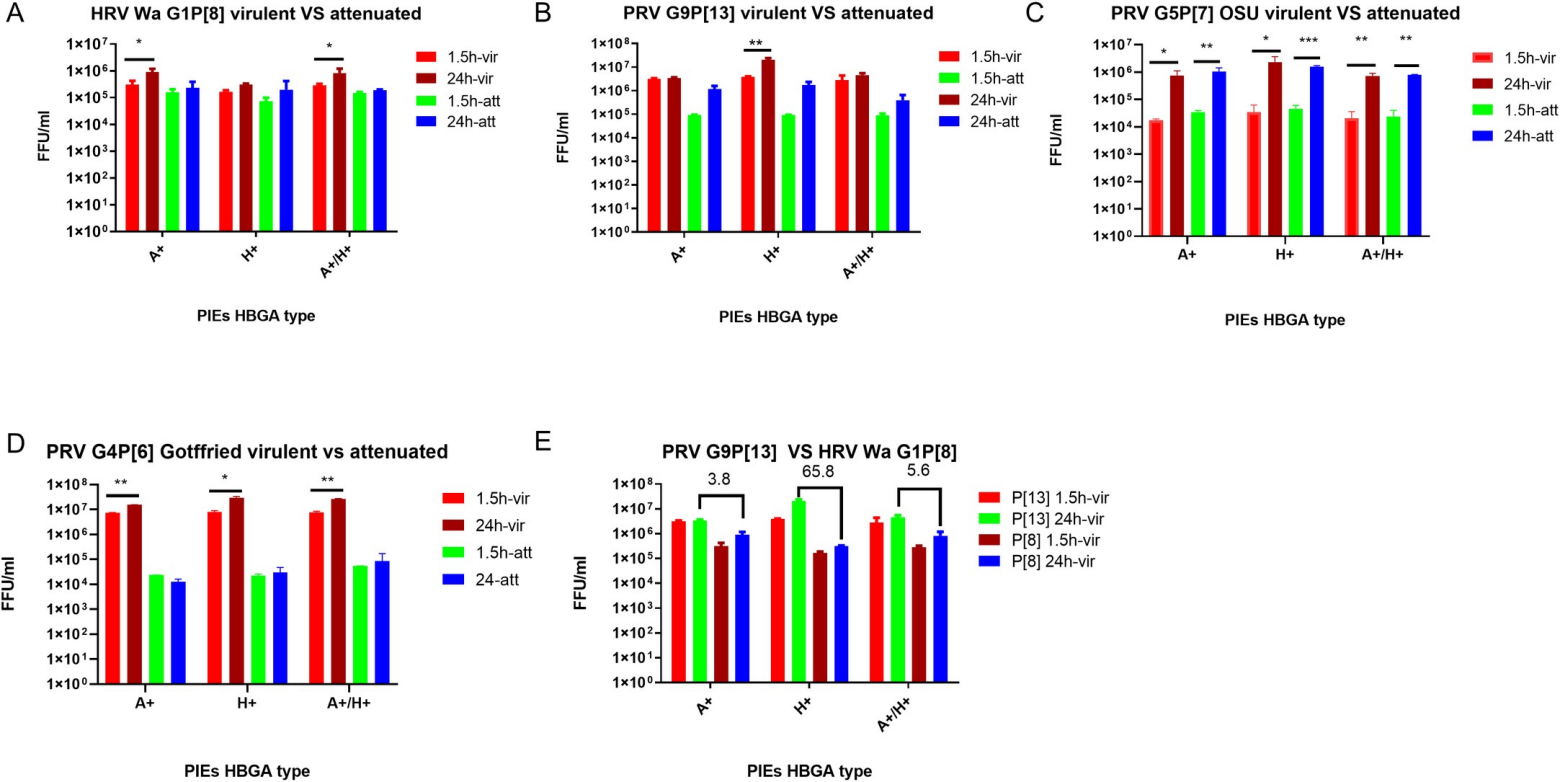
RV infectivity assay to determine the HBGA preference of different RV strains. RT-PCR infectivity in A+, H+, and A+H+ PIEs (x axis) for A) HRV Wa G1[P8] virulent vs attenuated B) PRV G9P[13] virulent vs attenuated C) PRV OSU G5P[7] virulent vs attenuated D) PRV Gottfried G4P[6] virulent vs attenuated. Ct values were converted to FFU/ml based on standard curve which combined CCIF and RT-PCR results (y axis). Red and green bars represent the virus titer of virulent and attenuated strains at 1.5hpi. Brown and blue bars represent the virus titer of virulent and attenuated strains at 24hpi. E) Virus titer comparison between HRV Wa and PRV G9P[13] virulent strains. The error bar represents the standard deviation from duplicate samples. All infectivity assays have been repeated at least at least 2–3 times. For P value: * p<0.05, ** p<0.01.

To evaluate the replication efficiency of RVs in PIEs with different HBGA types, differentiated PIEs were incubated with several virulent and attenuated RV strains including human G1P[8] Wa strain, porcine G4P[6] Gottfried strain, porcine G5P[7] OSU strain and porcine G9P[13] strains. RV levels were measured by RT-qPCR at 1.5 hours post-inoculation (hpi) and 24hpi. Our results indicated that virulent RVs replicated to higher titers in A+, H+ or A+H+ PIEs in a strain-specific manner (Fig 5 and Table 1). Specifically, virulent HRV G1P[8] Wa strain replicated to higher levels in the A+ PIEs (Fig 5A) while virulent PRV G9P[13], G5P[7] OSU and G4P[6] Gottfried strains replicated more efficiently in the H+ PIEs (Fig 5B–5D, respectively). Interestingly, most attenuated RV strains lost HBGA selectivity. However, the replication efficiency of the attenuated RVs varied for different strains. Attenuated PRV G9P[13] and PRV G5P[7] OSU strains replicated efficiently in PIEs (Fig 5B and 5C and Table 1) while attenuated HRV G1P[8] Wa and PRV G4P[6] Gottfried strains did not growth well (Fig 5A and 5D and Table 1). HRVs have been reported to grow better in HIEs than animal RV (ARV) [33]. Thus, we compared the replication efficiency of virulent PRV G9P[13] and HRV G1P[8] Wa strain to test if PRV would replicate better in PIEs than HRV. Our results showed that G9P[13] replicated to higher titers than G1P[8] Wa strain irrespective of HBGA types, and the fold change peaked (65.8 fold) in H type PIEs (Fig 5E). Overall, these results indicate that the replication efficiency of virulent RVs of different genotypes is significantly affected by the PIE HBGA types and suggest that homologous (PRV) replication may be more efficient than that of HRV in PIEs.

**Table 1:** Fold changes of RVs titers at 24hpi Fold changes were calculated by the virus titer at 24hpi / virus titer at 1.5hpi. vir: virulent strains att: attenuated strains.

Strain HBGA	Wa		G9P[13]		OSU		Gottfried	
	vir	att	vir	att	vir	att	vir	att
A+	2.9	1.4	1.1	12.5	42	31	2	0.5
H+	1.9	2.8	5.3	19	67	34	3.8	1.3
A+/H+	2.9	1.3	1.6	4.4	35	33	3.5	1.5

**Fig 6:**
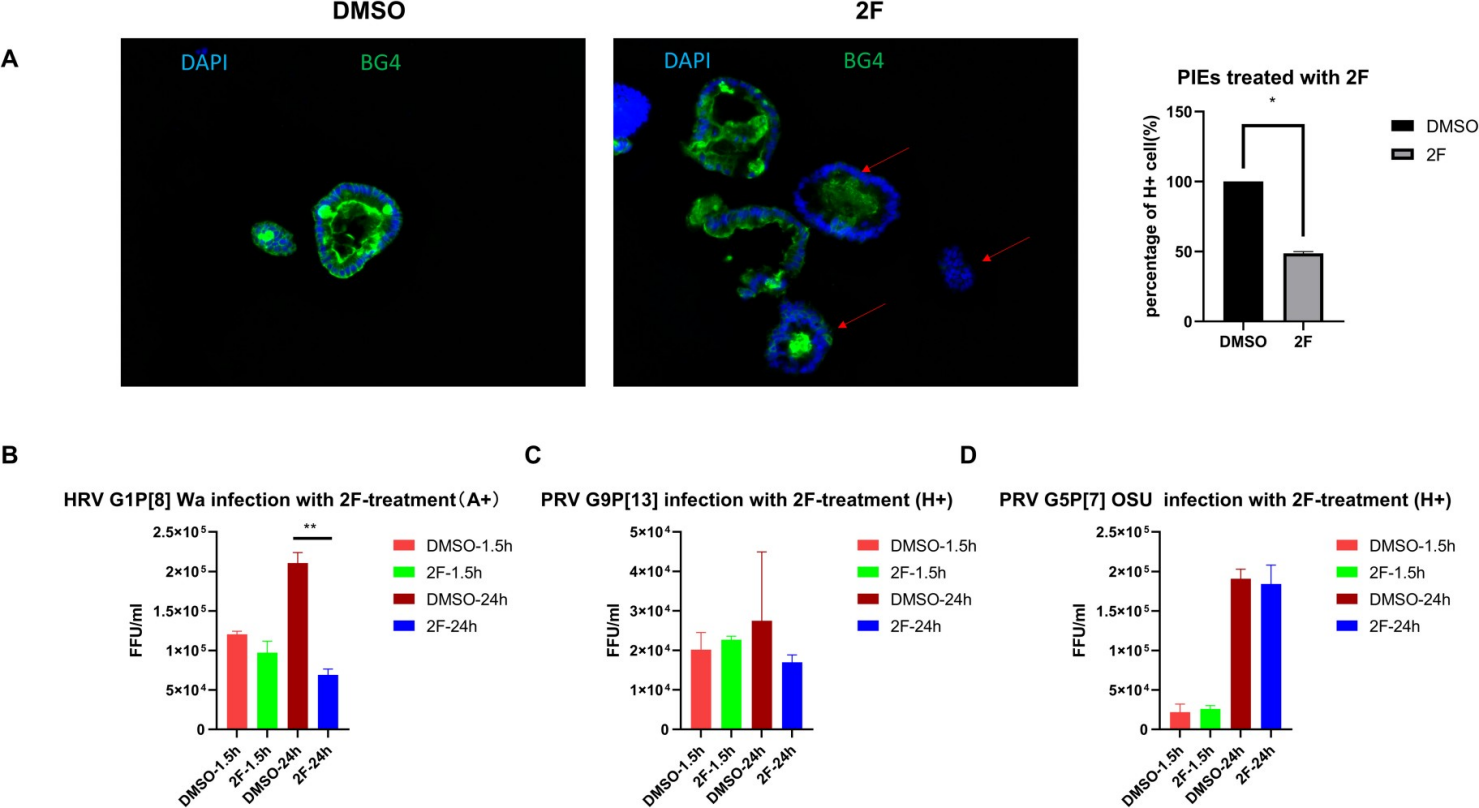
PIEs treated with 2F. 500 μM 2F or DMSO was added to differentiated PIEs for 3 days (refreshed medium and added new 2F or DMSO every day). Then 2F-treated PIEs were used either for A) IF detection of H antigen expression (BG4 antibody). BG4 (green) and DAPI (blue) staining are shown in a merged picture to denote the membrane staining pattern of H antigen. Left panel: H expression under DMSO treatment. Middle panel: H expression under 2F treatment. Red arrows denote PIEs with reduced H antigen expression. Right panel: Quantitive data for IF results. H+ and total cell numbers were counted and percentage of H+ cell s(%) are illustrated.

To further confirm the role of HBGAs in RV infection, a fucosyltransferase inhibitor, 2F-Peracetyl-Fucose (2F) [28,39,40] was used to inhibit the expression of HBGAs in the newly synthesized PIEs. After treating PIEs with 500 μM 2F (lacking cytotoxicity at this concentration) for 3 days, H antigen expression was tested by IF, and the result indicated that 2F could fully or partially block the H antigen expression on PIEs (Fig 6A, right panel). To test whether blocking of HBGA expression inhibits RV infection, 2F-treated A+ PIEs were infected with virulent Wa and 2F-treated H+ PIEs were infected with virulent OSU and G9P[13] strains. The infectivity assay showed that at 24hpi, Wa replication was decreased in 2F- treated A+ PIEs compared to the dimethyl sulfoxide (DMSO) treated control PIEs (Fig 6B). However, G9P[13] and OSU replication showed only a slight numeric decrease (not significant) between DMSO treated control and 2F-treated H+PIEs (Fig 6C and 6D, respectively). These results indicate that HBGAs may be important for HRV Wa infection; however, PRV OSU (SA-dependent strain) and G9P[13] may utilize HBGAs as alternative or co-receptors.

RT-PCR detection of B) Wa, C) G9P[13] and D) OSU infectivity in A+, H+, and H+ PIEs, respectively. Ct values were converted to FFU/ml based on a standard curve which combined CCIF and RT-PCR results. The error bar represents the standard deviation from duplicate samples. All experiments were repeated at least 2–3 times. For P values, **, p<0.01

### Contrasting roles of terminal SAs in RV replication

**Fig 7:**
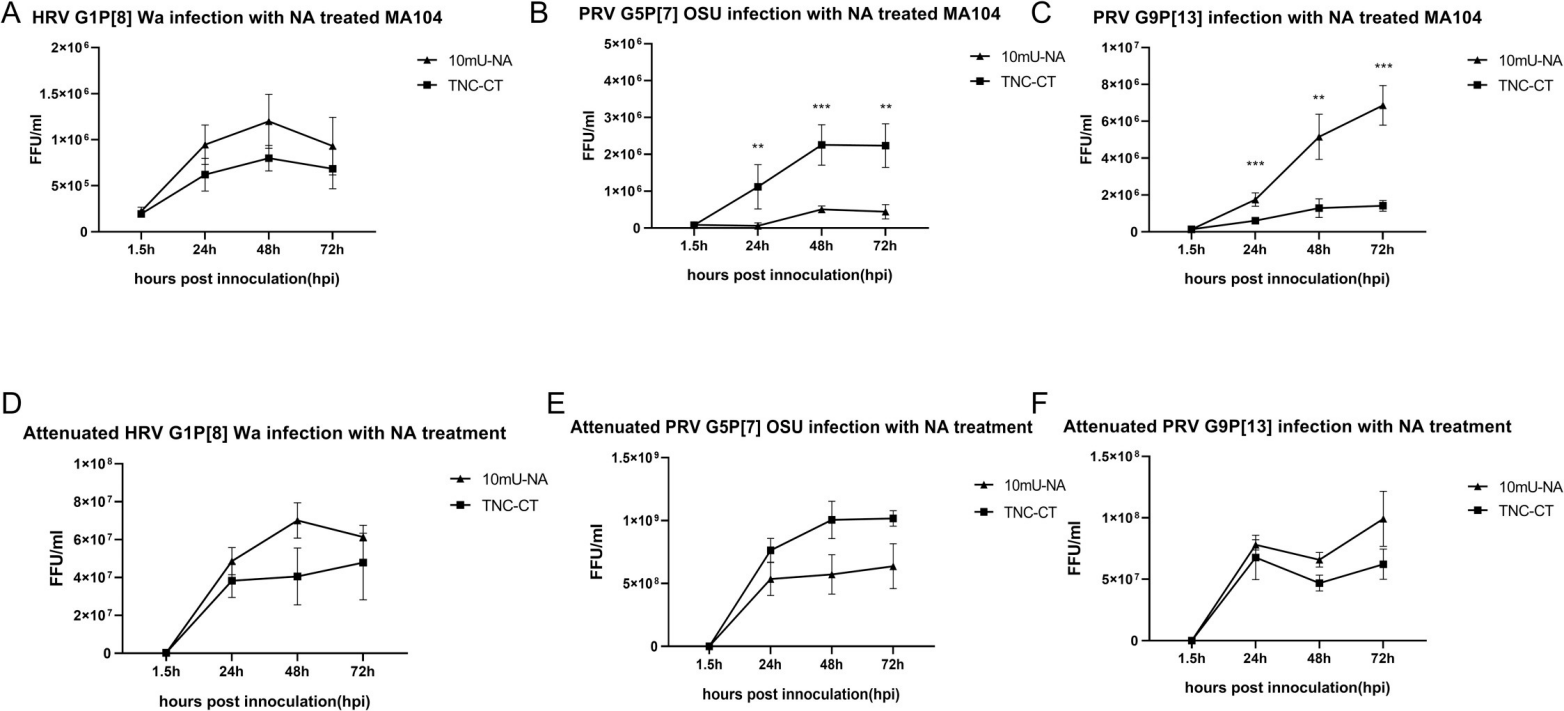
Sialidase-treatment of test RV strains. Growth curve of virulent A) Wa, B) OSU and C) G9P[13] after infection of sialidase treated MA104 cells, respectively. MA104 cells in 96 well plate were pre-treated with 10mU sialidase (Neuraminidase, NA) from *Arthrobacter ureafaciens* or TNC buffer (TNC-CT) for 1h at 37°C before inoculation. Then, MA104 cells were inoculated with 3000 FFU of RV and incubated for 1.5h at 37°C. Plates were harvested at 1.5hpi, 24hpi, 48hpi, 72hpi and the virus titers were measured by RT-PCR. Growth curve of attenuated D) Wa, E) OSU and F) G9P[13] after infection of sialidase treated MA104 cells. The inoculation process was the same for virulent RV infection as described above. The error bar represents the standard deviation from triplicate samples. All infectivity assays were repeated at least twice. For P value: * p<0.05, ** p<0.01, *** p<0.001.

**Fig 8:**
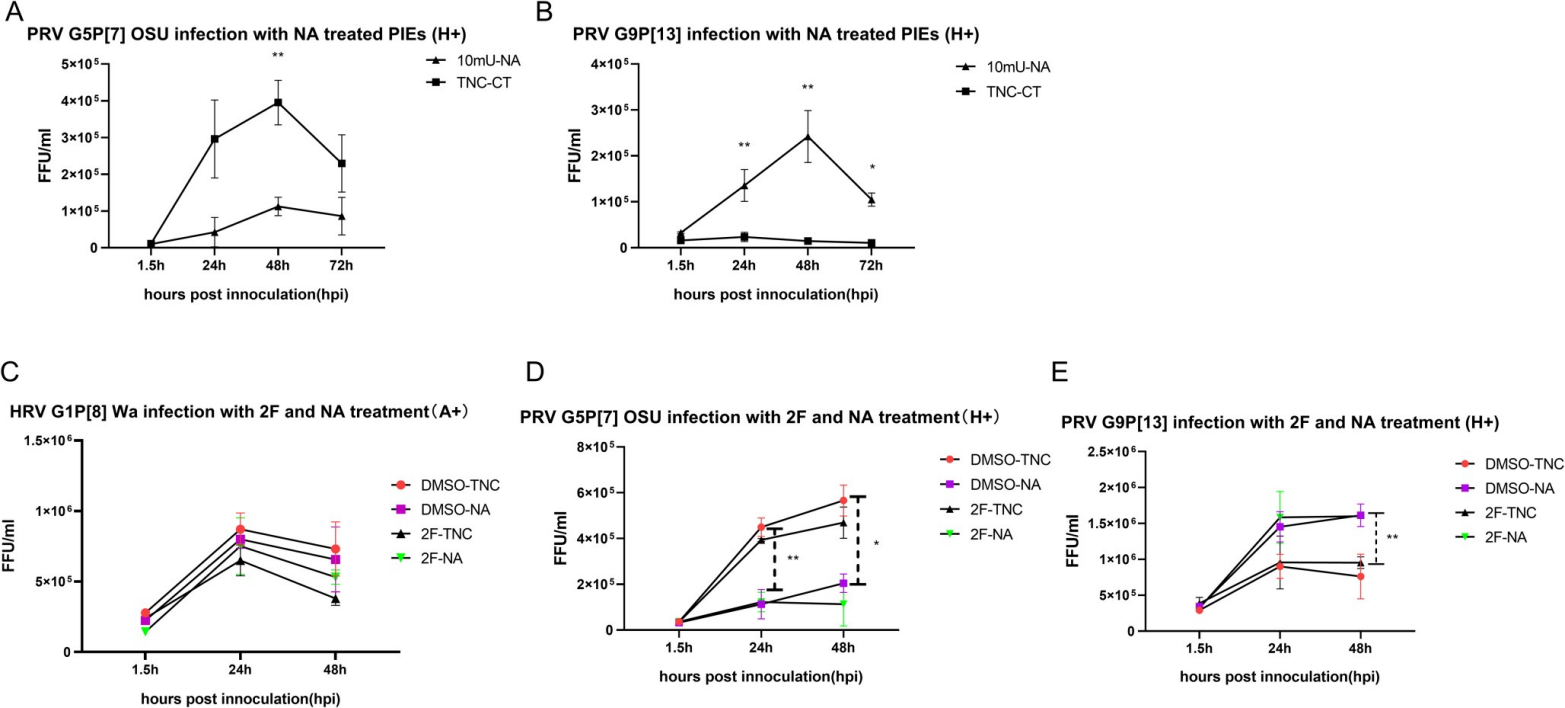
Sialidase and 2F double treatment on tested RV strains. Growth curves of A) OSU and B) G9P[13] in H+ PIEs treated with sialidase, respectively. PIEs were pre-treated with 10mU sialidase (10mU-NA) or TNC buffer (TNC-CT) for 1h at 37°C before inoculation. Then, PIEs were inoculated with RVs (MOI = 0.5) for 1.5h at 37°C. Plates were harvest at 1.5hpi, 24hpi, 48hpi, 72hpi and the virus growth were measured by RT-PCR. Growth curves of C) Wa, D) OSU and E) G9P[13] in A+ or H+ PIEs treated with sialidase and 2F. 500 μM of 2F or DMSO was added to differentiated PIEs for 3 days (the medium was replaced and 2F or DMSO were added daily). PIEs were then treated with 10mU-NA on the infection day followed by RV infection (MOI = 0.5). PIEs were re-plated into 3 96-well plates and harvested at 1.5hpi, 24hpi, 48hpi and the virus growth were measured by RT-PCR. Four different treatment groups were: DMSO-TNC (control group, represent by red circle), DMSO-NA (sialidase single treatment, represent by purple square), 2F-TNC (2F single treatment, represent by black triangle), and 2F-NA (2F and sialidase double treatment, represent by green triangle). The error bar represents the standard deviation from triplicate samples. All infectivity assays have been repeated at least twice. For P value: * p<0.05, ** p<0.01, *** p<0.001.

Because we only observed minimal effects of the 2F treatment on OSU and G9P[13] replication, we wanted to further explore the roles of SAs in their replication. OSU has been reported previously to be a sialidase-sensitive strain, while Wa and Gottfried were shown to be sialidase-insensitive strains [15]. Thus, we tested if SAs play a role during a PIE infection with G9P[13] whose sialidase sensitivity was unknown. Growth of Wa, Gottfried, OSU and G9P[13] were evaluated in MA104 cells that were pre-treated with 10mU of sialidase (denoted as 10mU-NA) or TNC buffer (contains 50 mM Tris-HCl pH 7.5, 150 mM NaCl, 10 mM CaCl2, 0.02% NaN3, denoted as TNC-CT). Consistent with previous reports, the growth of Wa and Gottfried strains was sialidase-insensitive or even showed a trend for a slight increase of infectivity following sialidase treatment (Figs 7A and S1, respectively), while replication of OSU was inhibited by sialidase treatment (Fig 7B). Interestingly, G9P[13] showed a reverse trend, whereby sialidase treatment significantly increased the G9P[13] growth in MA104 cell (Fig 7C). We further repeated the sialidase treatment for OSU and G9P[13] strains in our PIEs model, and the results demonstrated a trend similar to that observed in MA104 cells (Fig 8A and 8B, respectively).

Since all attenuated strains tested in this study did not demonstrate preference for certain HBGA types, we tested if NA sensitivity of these attenuated strains was also altered compared to their virulent counterparts. The resultant growth curves of attenuated Wa, OSU and G9P[13] (Fig 7D–7F, respectively) indicated that attenuated strains retained the same traits as their virulent counterparts. Specifically, NA treatment inhibited the growth of the attenuated OSU strain and enhanced the replication of attenuated Wa and G9P[13] strains, as was observed for the virulent Wa, G9P[13] and OSU strains. Interestingly, the inhibition or enhancement effects of NA treatment were less prominent in attenuated OSU or G9P[13] strains when compared with their virulent counterparts. Together, these results suggest that sialidase treatment strongly affects the replication of both OSU and G9P[13] strains, but in contrasting ways.

To further clarify the role of HBGAs and SAs in RV replication, a combined NA-2F treatment was performed for Wa, OSU and G9P[13] strains. A+ (for Wa infection) or H+ (for OSU and G9P[13] infection) PIEs were divided into four treatment groups: DMSO-TNC (control group), DMSO-NA (sialidase single treatment group), 2F-TNC (2F single treatment group), and 2F-NA (2F and sialidase double treatment group). The growth of Wa (Fig 8C) did not differ significantly among the 4 groups at 3 different time points. However, consistent with our previous observations, a numeric reduction in Wa titer was was evident in the 2F treated PIEs at 24hpi and 48hpi when compared with the DMSO-TNC with 2F-TNC treated ones. In contrast, NA treatment did not affect Wa growth in the DMSO-TNC and DMSO-NA groups, and only slightly enhanced Wa replication between the 2F-TNC and 2F-NA groups, likely by counteracting the effects of 2F treatment. These results suggest that HBGA may play a more important role than SAs in Wa infection of PIEs. A different scenario was observed for OSU strain (Fig 8D), where sialidase treatment significantly reduced the virus titer at 24hpi and 48hpi in DMSO-NA vs. DMSO-TNC treated PIEs, as well as in 2F-NA vs. 2F-TNC treated PIEs. Noticeably, 2F treatment had no effect (24hpi) or only slightly inhibited OSU growth (at 48hpi) in 2F-TNC/2F-NA vs. DMSO-TNC/DMSO-NA treated PIEs. Together, these results indicate that the inhibitory effect of the NA treatment is more pronounced during OSU infection than that of the 2F treatment. Finally, the G9P[13] (Fig 8E) replication profile indicated that NA dependent replication enhancement was significant at 48hpi while 2F treatment had no or marginal effects on the growth of G9P[13]. These results suggest the pivotal role of internal SAs (or some other factors being unmasked by NA treatment) in G9P[13] infection when compared with HBGAs [41]. Together, these experiments also highlight contrasting mechanisms of interactions with SAs employed by PRV OSU and G9P[13] strains in the corresponding host cells. Importantly, the double (2F-NA) treatment did not result in complete inhibition of replication of any of the RV strains tested in this study, suggesting that additional cellular receptors/attachment factors (such as internal SAs) may be utilized by most RV strains.

## Discussion

HBGAs have been reported as potential receptors or attachment factors for RVs [12,17,24–28,42–44]. In the present study, we evaluated the replication of several RVA strains prevalent in humans (G1P[8] Wa strain) or swine (G4P[6] Gottfried, G5P[7] OSU and G9P[13] strains) in PIEs. Our results strongly suggest that virulent RVs are capable of selective interactions with different HBGAs expressed by PIEs. Our results showing that G1P[8] Wa replicated to the highest titers in A+ PIEs, while G4P[6] Gottfried replication was only slightly higher in H+ and A+H+ PIEs vs. A+ are in agreement with the previous findings that P[6] and P[8] RVs could bind to type 1 precursor and ABH antigens [12,27]. In contrast, G5P[7] OSU and G9P[13] strains showed a strong preference for the H+ PIEs. P[7] and P[13] both belong to the P[I] group of RV [15,24]. Our results suggest that P[I] PRVs may selectively recognize H type1 HBGA and the receptors for RV entry may be redundant since the OSU strain is both SA and H dependent. These observations may provide one explanation for the remarkably robust replication of the OSU strain in most in vitro and in vivo models [45].

Live attenuated oral vaccines like Rotarix (GlaxoSmithKline, GSK) provide good protection to infants and children in developed countries but perform poorly in the developing world [46–49]. The ability of RV VP4 to selectively interact with the host HBGAs could potentially lead to variable vaccine efficiency and inconsistent performance in individuals with different HBGA blood groups. To address this concern, we compared attenuated vs. virulent RV strains and confirmed that consistent with the absence of HBGA expression by MA-104 cells, most attenuated RVs lose HBGA selectivity. Attenuated Wa and Gottfried strains show decreased replication ability in the PIEs while attenuated G9P[13] and OSU strains replicated quite efficiently in PIEs (Fig 5 and Table 1), which may be indicative of variable of attachment efficiency of attenuated strains. The underlying reasons for the loss of HBGA selectivity during attenuation remain unknown; however, it could result from mutations in the receptor binding domain of the VP4 of attenuated RV strains (for example, D393H in OSU and D385N in other RV strains) [50]. It is also consistent with previous observations that live attenuated RV vaccines have relatively broad protection among the population [51–53] which means vaccine (cell culture adapted = attenuated) strains may have lost their preference for a certain HBGA. One possible molecular determinant of the unusually efficient replication of attenuated OSU in PIEs could be our recent finding of a conserved D385N mutation in the VP4 of the attenuated Wa, Gottfried [50] and G9P[13] strains, but not in the attenuated OSU strain. Since the D385N mutation is located near the VP5* hydrophobic loop and may affect virus entry, we hypothesize that the D385N mutation could be associated with the reduced replication of most attenuated RVs in PIEs/pigs [54]. However, the specific function of the D385N mutation needs to be determined in further studies using reverse genetics [55,56]. We also observed that the virulent PRV G9P[13] strain replicated better in PIEs than HRV Wa strain regardless of the HBGA type (Fig 5E). This agrees with previous observations that HRV replicate to higher titers in HIEs than animal RVs [33] and our previous findings that porcine RVs replicate better than human RVs in Gn pigs [57]. One explanation for the reduced growth of heterologous RVs in IEs could be the incomplete homology of HBGA between humans and swine. Additionally since RVs have also been proposed to interact with the heat shock cognate protein 70 (hsc70) and integrin for entry after initial attachment, incomplete homology of these proteins shared between different species could be another reason [58].

Consistent with our results, an epidemiological study showed that among children admitted to hospitals with RV gastroenteritis (Valencia, Spain), those secretor+ with A or AB blood group types were significantly more prone to acute gastroenteritis caused by the prevalent P[8] RV strains [59]. Although several studies support the correlation between secretor status and P[8] HRV infection resistance [59–62], other evidence indicates that P[8] HRV could infect both secretor positive and negative individuals [33, 63]. A possible explanation for the variable results generated in these studies could be: 1) Sample size limitation; 2) FUT2 polymorphism in different population; 3) Predominant RV genotype differences among the regions studied. Thus, further investigation is required to understand the associations between secretor status and RV infection resistance.

Our sialidase treatment results confirmed previous observations demonstrating that replication of sialidase-sensitive strains, like G5P[7] OSU, was significantly inhibited by sialidase treatment, while sialidase treatment showed no inhibition or only a slight increase in the growth of sialidase-insensitive strains like G1P[8] Wa [15,64]. Surprisingly, sialidase treatment resulted in significant enhancement of G9P[13] replication instead of inhibition. Interestingly, attenuated strains retained the key traits of their virulent counterparts after sialidase treatment. However, the overall effect of sialidase treatment was diminished which is similar to the observation that attenuated strains have lost HBGA selectivity (Fig 5). These results may indicate the ability of RVs to recognize the original receptors is decreased during the attenuation process. When HBGA synthesis was inhibited by 2F treatment, HRV Wa but not PRV OSU or PRV G9P[13] growth was decreased. Together with 2F-sialidase double treatment, these results suggest that: 1) Although HBGA types affect the growth of both HRV and PRV strains, they are critical for Wa infection of PIE, but appear to be less essential for OSU and G9P[13]. Thus, we conclude that HBGAs may serve as co-factor or alternative receptors for these PRV strains; 2) some unknown factors masked by terminal SA may be essential for G9P[13] attachment and replication[41]. Glycans that contain internal SA may be the potential target to investigate the mechanisms of the enhanced replication of G9P[13] after sialidase treatment. Whether SA interact in concert with HBGAs or represent alternative routes for RV entry remains to be determined. Finally, we plan to design and conduct in vitro and animal studies to determine what NA sources G9P[13] may utilize to enhance its replication in vivo, and whether this mechanism could contribute to its increasing global prevalence.

Collectively, we have successfully established PIEs expressing major porcine HBGA types providing, a physiologically functional in vitro system to comprehensively evaluate RV-host glycan interactions. Our RV infection results strongly support the original hypothesis that replication of virulent RVs is significantly affected by HBGA types expressed on PIEs and this HBGA selectivity is strain dependent. Further, this study demonstrate that the efficacy of live attenuated RV vaccines may not be affected by variable host HBGA phenotypes, because attenuation via cell culture adaptation may reduce RV HBGA selectivity. Furthermore, we concluded that removal of terminal SAs may not only inhibit (as was previously demonstrated), but can also greatly enhance attachment and/or replication of some RV strains. Finally, this research broadens our knowledge on SA-HBGA-RV interactions and provides novel insights into RV evolutionary mechanisms potentially critical for its epidemiology, prevalence, zoonotic transmission, and development of preventative and interventional strategies.

## Materials and methods

### Ethics statement

All of the pigs were derived, maintained, and euthanized as described in the protocol #2010A00000088 (approved by OSU IACUC) in accordance with guidelines of public health service policy on humane care and use of lab animal and USDA animal awareness act guideline for animal care and use of lab animal (Public Health Service, 2002 and US Department of Agriculture, 1985). The pigs were euthanized using Telazol-Ketamine-Xylazine intra-muscular injection for anesthesia which was followed by intracardiac injection of pentobarbital.

### Rotavirus strains

Pairs of virulent and cell-culture adapted (attenuated) human/animal group A RVs used: human G1P[8] Wa strain, porcine G4P[6] Gottfried strain, porcine G5P[7] OSU strain, and G9P[13] RV0084 strain. The virulent strains were serially passaged in gnotobiotic (Gn) pigs for 9 (Gottfried), 12 (OSU), 22 (Wa) and 5 (G9P[13]) passages as described [45, 65], and their attenuated counterparts were serially passaged in MA-104 cell culture for 54 (Gottfried), 43(OSU), 28 (Wa) and 54 (G9P[13]) consecutive passages as described [66]. The attenuation status of the cell-culture adapted variants was previously confirmed in Gn piglets as described [45,65]. Virulent and attenuated G1P[8] Wa, G4P[6] Gottfried and G5P[7] OSU strains have been sequenced and detailed information is presented in our recent paper [50]. The NCBI accession number of the VP4 genes are as follows: Wa: MT025868, MT025869; Gottfried: MT025912, MT025913; OSU: MT025934, MT025935.

### Enteroid establishment, propagation, passaging and differentiation

Media used for PIE establishment, maintenance and differentiation were formulated as follows: complete medium without growth factors [CMGF(-) medium] consisted of advanced DMEM/F12 medium (Invitrogen) supplemented with 100 U/ml penicillin-streptomycin (Invitrogen), 10mM HEPES buffer (Invitrogen), and 1X GlutaMAX (Invitrogen) [30,33]. Complete cell culture medium (CCCM) contained IntestiCult Organoid Growth Medium (Component A: Component B = 1:1, STEMCELL), 100 U/ml penicillin-streptomycin (Invitrogen) and 2.5 μm CHIR99021 (Stemgent). Per the manufacturer’s recommendations, differentiation medium (DM) should have contained IntestiCult Organoid Growth Medium Components A and B added to the ratio of 1:0.5, supplemented with 100 U/ml penicillin-streptomycin (Invitrogen). However, this ratio resulted in complete growth inhibition and unsatisfactory viability of the PIEs; therefore, we have explored additional ratios as described in the Results.

Crypt cells were extracted from the small intestine (ileum) as described by Sato et al. with some modification [30]. Briefly, approximately 5–8 cm of anterior ileum was collected from 3-week-old healthy Gn piglets (Yorkshire White x Landrace x Duroc). After opening the intestinal segment longitudinally, the tissue was washed 4–5 times in ice cold Dulbecco's Phosphate Buffered Saline (DPBS, Gibco Life Technologies), stretched and secured with the mucosal side facing up on a silicone-coated glass Petri dish filled with ice-cold DPBS using minutien pins. Under dissecting microscope, the overlying mucosa was separated from the submucosa and connective tissue using micro-dissecting scissors and fine point curved forceps. Then the surface of the mucosa was gently scraped with curved forceps to remove the villi. Wash the mucosa 3–4 times with ice-cold chelation buffer to remove villi and debris. Then the mucosa was overlaid with freshly prepared 2mM EDTA chelation buffer (200 μl 0.5 M EDTA in 49.6 ml chelation buffer) and shaken gently for 30 min on a horizontal orbital shaker. After washing 3–4 times with ice-cold chelation buffer without EDTA, the tissue was left in ice-cold chelation buffer and gently scraped under dissecting microscope using curved and fine forceps release intestinal crypts. The crypt suspension was collected into a 50 ml conical tube, filtered through a 150 μm mesh 2 times and centrifuged for 5 min at 150 x g, at 4°C. The pellet was resuspended in 5 ml ice-cold chelation buffer and the crypts were counted. For three-dimensional PIEs culturing, 50 crypts will be mixed with 25 μL of Matrigel (clear, Corning, USA) and seeded in a 48-well plate pre-warmed at 37°C, 50 crypts/well. After solidification of Matrigel 350 μL of IntestiCult Organoid Growth Medium (Human) (STEMCELL Technologies) were added and placed at 37°C, 5% CO2 incubator, and the medium was changed every other day. For the passage of the PIE, 1 mL of Gentle Cell Dissociation Reagent (GCDR, STEMCELL) was added after the medium is removed. Enteroids were pipetted vigorously and broken down into small pieces by using 25G needle and syringe. Those pieces were pelleted by centrifuging at 400 × g for five minutes at 4°C and then mixed with Matrigel for plating.

### Immunofluorescence (IF) antibody staining for HBGA typing

PIEs were washed using 1X phosphate buffered saline (PBS) at room temperature (RT) after removing CCCM and then fixed with 1ml of 2% paraformaldehyde (PFA) 0.1% glutaraldehyde (GA) in 1X PBS for 30 minutes, RT. Fixed PIEs were washed with 1X PBS 3 times for 10min. After washing, Matrigel domes were collected using a scoop and placed in a 50 ml conical tube containing 20% sucrose in 1X PBS, and the tube was left at 4°C until the domes fall to the bottom of the tube (overnight or longer as needed). Once the domes were in the bottom of the tube, sucrose solution was removed, and the domes were placed in a mold followed by adding Tissue-Tek O.C.T. compound (Sakura) for embedding. Molds were snap- frozen and stored at -80°C until use.

For sectioning, a cryotome (LEICA CM 1510S) was used and PIE blocks were cut in approximately 5 or 8 um thick and heat-mediated antigen retrieval was performed on PIE sections using 1X reveal decloaker (BIOCARE). Slides were washed using 1X PBS and treated with 0.15% Triton to increase the permeability of the cell membrane. Then slides were washed again using 1X PBS and blocking was performed using 3% Bovine Serum Albumin (BSA). The following primary antibodies were diluted in 3% BSA: mouse BG-2 mAb (1:200, Biolengend, anti-A), mouse BG-4 mAb (1:200, Biolengend, anti-H type 1), rabbit SOX9 (1:500, Millipore). The slides were incubated with the diluted primary antibodies at 4°C, overnight, and washed using 1x PBS. Then the following secondary antibodies diluted in 3% BSA were added to the respective slides: Alexa Fluor 488 Goat anti-mouse IgG3 (1:1000, Thermo Fisher Scientific), Alexa Fluor 488 Goat anti-rabbit IgG (1:1000, Thermo Fisher Scientific). All secondary antibodies were incubated with slides at RT temperature for 2h. DAPI (4′,6-diamidino-2-phenylindole) was used for nuclear staining and Vector TrueVIEW Autofluorescence Quenching Kit was used to reduce background fluorescence. Slides were sealed by adding mounting medium and pictures were using Keyence BZ-810 microscope.

### RV infection

To achieve the correct multiplicity of infection (MOI), 1ml of accutase cell dissociation solution (BD Biosciences) was added to each well and incubated at 37°C for 30min. The cell number was counted by Cellometer Auto T4 (Nexcelom Bioscience). The MOI was calculated as the amount of input virus/total number of cells in samples of PIEs. For RVs infection, PIEs were differentiated in DM for at least 4 days. GCDR was used to remove matrigel and an apical exposure to RV was performed using 200μl pipette as previously described [33]. The RV inocula were pre-activated using 10ug/ml trypsin for 30min at 37°C and diluted by loading solution which contains CMGF- and 0.25mg/ml pancreatin to achieve a desired MOI (set at 0.5 unless specified). PIEs were incubated with the RV inocula for 1.5h at 37°C and then washed twice using cold CMGF- medium and resuspended in an appropriate amount of loading solution. 100μl of PIEs were plated in 96-well plates in duplicates and plates were harvested at certain timepoints and frozen at -80°C until use.

### 2F-Peracetyl-Fucose and sialidase treatment

2F-Peracetyl-Fucose is a cell-permeable fluorinated fucose derivative that acts as an inhibitor of fucosyltransferases following its uptake and metabolic transformation into a GDP-fucose mimetic[28,39,40]. For 2F-Peracetyl-Fucose (2F, Sigma-Aldrich) treatment, 500 μM 2F or DMSO (control) was added to differentiated PIEs for 3 days (refresh medium and add new 2F or DMSO every day) and PIEs were infected by RVs as described above. Cell viability was examined before infection and no obvious differences were observed between DMSO and 500 μM 2F treated PIEs. For sialidase treatment, MA104 cells in 96 well plate or PIEs were pre-treated with 10mU sialidase from *Arthrobacter ureafaciens* (Roche) for 1h at 37°C before inoculation. TNC buffer (50 mM Tris-HCl pH 7.5, 150 mM NaCl, 10 mM CaCl2, 0.02% NaN3) were used to dilute sialidase and serve as a control for sialidase treatment. Following 2F or sialidase treatment, PIEs were infected by RVs as described above.

### RNA/DNA extraction and PCR/qRT-PCR

A lysis buffer containing 0.5mg/ml protease K (Amresco), 50 mM KCl (Sigma), 10 mM Tris-Cl pH 8.0 (Invitrogen), 2.5 mM MgCl2 (Sigma), 0.45% IGEPAL (Sigma), 0.45% Tween-20 (Promega) and DEPC-treated water (Promega) was used for total RNA extraction. Briefly, 200μl of lysis buffer was added in each well in 96-well plate that contains PIEs and was incubated for 30min at 37°C. Then 100μl of the PIEs-lysis buffer mixture was transferred to a 96-well PCR plate followed by incubating in a PCR cycler and using the following parameters: 30min at 65°C, 2min at 98°C and cool down to 4°C. The RNA concentration was tested by Nanodrop and adjusted to the same level. Power SYBR Green RNA-to-CT 1-Step Kit (Thermo Fisher) was applied for Quantitative reverse transcription-PCR (qRT-PCR) according to the manufacturer’s protocol. qRT-CR to measure RV RNA levels was conducted as previously described [57,67]. For SOX9, PCNA and MUC2 genes, mRNA expression levels were normalized to β-actin mRNA levels using the 2^-ΔΔCT^ method as previously described [68]. qRT-PCR results were graphed using GraphPad Prism software. To establish PIE cultures with major HBGA types (A+, H+, and A+/H+) in porcine, Gn piglets that are 1 week old were AHO genotyped using qRT-PCR and PCR methods to confirm fucosyltransferase 2 (FUT2) mRNA expression and erythrocyte antigen A (EAA) locus presence, respectively, as described [38]. Due to polymorphisms in the FUT2 and EAA genes, the presence of both functional genes may result in either A+, A+H- or A+/H+ phenotypes of the gut epithelial cells (See S1 Table, E4P5) [37,38].

### Statistical analyzes

GraphPad Prism v 5.0 (GraphPad Software, San Diego, CA, USA) were used for data analysis. Comparison of the quantified virus replication data between different treatments was done using Student t-test unless specified. Differences were considered statistically significant when p ≤ 0.05.

## Supporting information

S1 Fig Sialidase-treatment of MA104 cells to determine NA sensitivity of PRV Gottfried strain. Growth curve of virulent Gottfried strain after infection of sialidase treated MA104 cells. MA104 cells in a 96 well plate were pre-treated with 10mU sialidase (Neuraminidase, NA) from *Arthrobacter ureafaciens* or TNC buffer (TNC-CT) for 1h at 37°C before inoculation. Then, RVs with 3000 FFU were inoculate with MA104 cells for 1.5h at 370C. Plates were harvest at 1.5hpi, 24hpi, 48hpi, 72hpi and the virus growth were measured by RT-PCR.
(TIF)

S1 Table PIE genotyping and phenotyping by erythrocyte antigen A (EAA) PCR and IF staining, respectively. This table shows the genotyping and phenotyping results of the established PIEs. EXPX indicates experiment and pig numbers, respectively. E3P12, E4P5 and E4P13 were used in this study; however, the results were reproducible in PIEs from different pigs expressing the same HBGAs.
(DOCX)
