# Supplementary figures and images for: Infection of porcine small intestinal enteroids with human and pig rotavirus A strains reveals contrasting roles for histo-blood group antigens and terminal sialic acids
Source: PLoS Pathog. 2021 Jan 29;17(1):e1009237. doi: 10.1371/journal.ppat.1009237 (PMC7846020; doi:10.1371/journal.ppat.1009237)

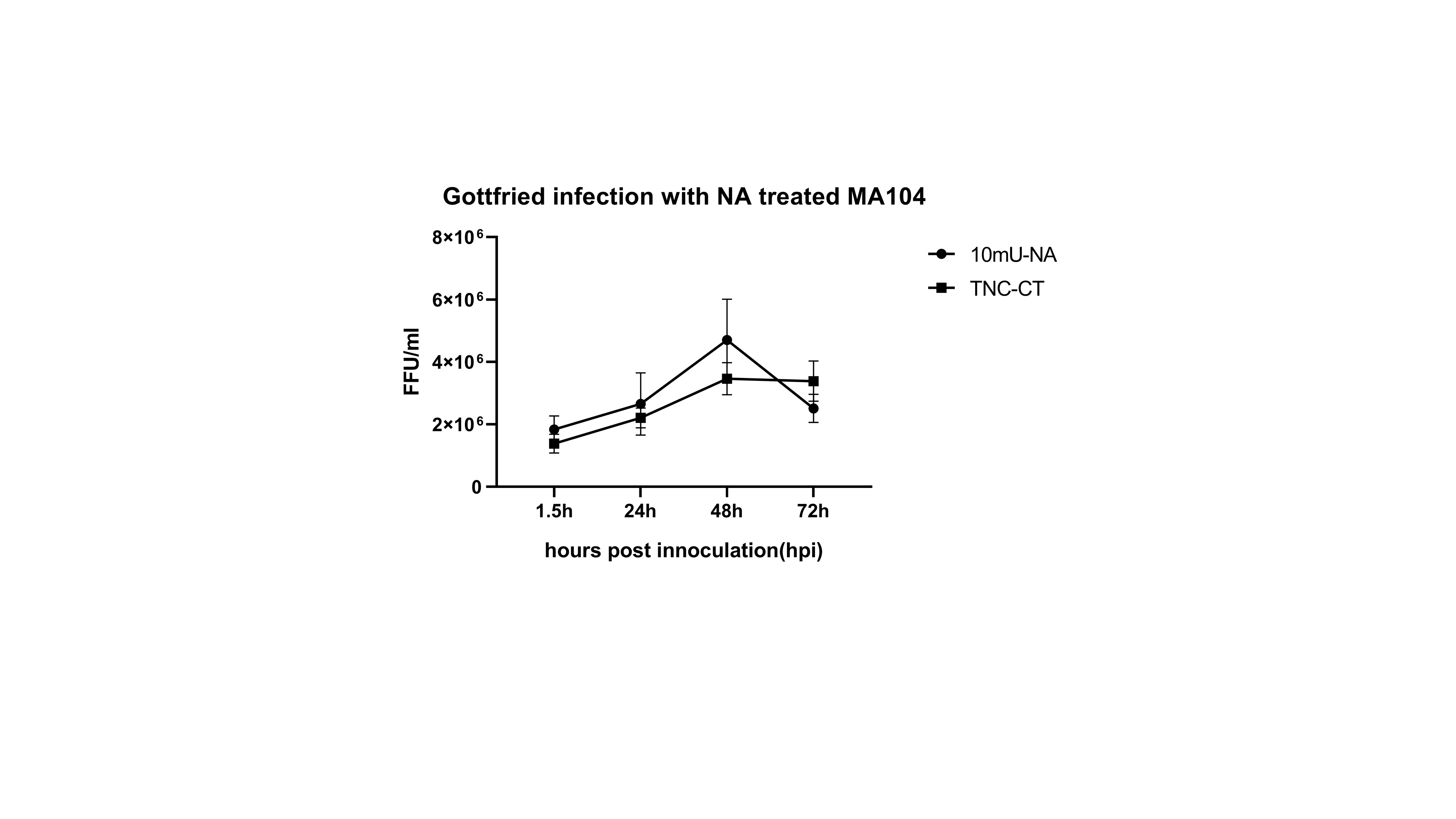

Supplement: S1 Fig — Growth curve of virulent Gottfried strain after infection of sialidase treated MA104 cells. MA104 cells in a 96 well plate were pre-treated with 10mU sialidase (Neuraminidase, NA) from Arthrobacter ureafaciens or TNC buffer (TNC-CT) for 1h at 37°C before inoculation. Then, RVs with 3000 FFU were inoculate with MA104 cells for 1.5h at 370C. Plates were harvest at 1.5hpi, 24hpi, 48hpi, 72hpi and the virus growth were measured by RT-PCR. (TIF) [file ppat.1009237.s001.tif]
