# Supplementary material for: Infection of porcine small intestinal enteroids with human and pig rotavirus A strains reveals contrasting roles for histo-blood group antigens and terminal sialic acids
Source: PLoS Pathog. 2021 Jan 29;17(1):e1009237. doi: 10.1371/journal.ppat.1009237 (PMC7846020; doi:10.1371/journal.ppat.1009237)
Supplement: S1 Table — This table shows the genotyping and phenotyping results of the established PIEs. EXPX indicates experiment and pig numbers, respectively. E3P12, E4P5 and E4P13 were used in this study; however, the results were reproducible in PIEs from different pigs expressing the same HBGAs. (DOCX) [file ppat.1009237.s002.docx]

|  | IF staining | EAA PCR |
| --- | --- | --- |
| E3P10 | A+H+ | A+H+ |
| E3P11 | A+H+ | A+H+ |
| E3P12 | A-H+ | A-H+ |
| E3P13 | A-H+ | A-H+ |
| E4P2 | A+H+ | A+H+ |
| E4P5 | A+H- | A+H+ |
| E4P13 | A+H+ | A+H+ |
